# Short version of the “instrument for assessment of stress in nursing students” in the Brazilian reality

**DOI:** 10.1590/1518-8345.2071.2976

**Published:** 2018-01-08

**Authors:** Ana Lúcia Siqueira Costa, Rodrigo Marques da Silva, Fernanda Carneiro Mussi, Patrícia Maria Serrano, Eliane da Silva Graziano, Karla de Melo Batista

**Affiliations:** 1 PhD, Associate Professor, Escola de Enfermagem, Universidade de São Paulo, São Paulo, SP, Brazil.; 2 Doctoral student, Escola de Enfermagem, Universidade de São Paulo, São Paulo, SP, Brazil. Scholarship holder at Conselho Nacional de Desenvolvimento Científico e Tecnológico (CNPq), Brazil.; 3 PhD, Associate Professor, Departamento de Enfermagem, Universidade Federal da Bahia, Salvador, BA, Brazil.; 4 MSc, Professor, Pontifícia Universidade Católica, Sorocaba, SP, Brazil.; 5 PhD, Adjunct Professor, Departamento de Enfermagem, Universidade Federal de São Carlos, São Carlos, SP, Brazil.; 6 PhD, Adjunct Professor, Departamento de Enfermagem, Universidade Federal do Espírito Santo, Vitória, ES, Brazil.

**Keywords:** Nursing, Students, Nursing, Stress, Psychological, Psychometrics

## Abstract

**Goal:**

validate a short version of the Instrument for assessment of stress in nursing students in the Brazilian reality.

**Method:**

Methodological study conducted with 1047 nursing students from five Brazilian institutions, who answered the 30 items initially distributed in eight domains. Data were analyzed in the R Statistical Package and in the latent variable analysis, using exploratory and confirmatory factor analyses, Cronbach’s alpha and item-total correlation.

**Results:**

The short version of the instrument had 19 items distributed into four domains: Environment, Professional Training, Theoretical Activities and Performance of Practical Activities. The confirmatory analysis showed absolute and parsimony fit to the proposed model with satisfactory residual levels. Alpha values ​​per factor ranged from 0.736 (Environment) to 0.842 (Performance of Practical Activities).

**Conclusion:**

The short version of the instrument has construct validity and reliability for application to Brazilian nursing undergraduates at any stage of the course.

## Introduction

In the process of undergraduate nursing training, academic, clinical and socioeconomic factors can impact the academic performance and health of students. The overload of theoretical courses, the level demanded in tests and extra-class activities, the interpersonal relationship with teachers and the fear of not succeeding are common aspects of the academic context^1^ ^-^ ^3^. In the field of care, students witness the suffering and death of patients, they lack technical ability and knowledge for clinical practice and need to interact with the health team^1^ ^-^ ^3^. In addition, they often spend considerable time to travel to the university and/or field of study; they face financial difficulties to ensure the personal cost of living and the course itself; and need to reconcile academic life with social and family activities^1^ ^-^ ^4^. These situations can be perceived as overwhelming and beyond their coping capacity and lead to neuroendocrine manifestations of stress. 

This phenomenon impacts the students’ health, with possible loss in academic performance^1^, increased risk of depressive symptoms^5^ and Burnout Syndrome^6^ ^-^ ^7^. Research carried out with 88 nursing students in São Paulo identified a statistically significant correlation between stress levels and the occurrence of depressive symptoms^5^. The correlation between stress and the Burnout Syndrome was demonstrated in a study involving 75 undergraduate nursing students from the United States^6^ and 161 dentistry students from Switzerland^7^. In this context, it is necessary that stress factors be correctly assessed through valid and reliable instruments. The international literature has some instruments to assess stress in general populations^8^ ^-^ ^9^, which have also been applied to university students^8^ ^-^ ^9^. In Brazil, the instrument for Assessment of Stress in Nursing Students (ASNS) was created in 2009 with 30 items organized in six explanatory factors^4^ ^,^ ^10^ ^-^ ^11^. 

The ASNS aims to measure the occurrence of stress factors in different academic contexts and has already been applied in several places in the Brazilian territory. However, the application of assessment instruments with less items has some benefits such as shorter time of application, better adherence of participants, and lower risk of filling induced by tiredness, especially when the instrument has a large number of items^12^ ^-^ ^13^. Short instruments also increase the explanatory power of the set of the remaining variables and enables the identification of subgroups that evaluate the same cognitive ability or capacity (factors, domains, dimensions or components)^14^. In view of these benefits and the frequent use of the ASNS in Brazil, the present study had the aim to validate a short version of the Instrument for assessment of stress in nursing students in the Brazilian reality.

## Method

This is a methodological study carried out with five Brazilian higher education institutions located in different regions of the country. Students enrolled in Nursing Undergraduate Courses, from the 1st to the 8th semester, of both sexes, and aged 18 years or over were included in the study. Students not enrolled in subjects of the professional cycle who had not completed the curriculum because they had exceeded the time limit of each school, and students who were not present on the day of data collection and who were in exchange training during the period of data collection were excluded from the study. Students were approached in the classroom in previously scheduled times, as agreed with the teacher of the subject, and they were also individually located when necessary. 

Data was collected at different periods in each institution, from April 2011 to March 2016, through the application of the Instrument for Assessment of Stress in Nursing Students (ASNS)^2^. This instrument is composed of 30 items grouped in six domains: Performance of Practical Activities (Items 4,5,7,9,12 and 21); Professional communication (Items 6,8,16 and 20); Time management (Items 3,18,23, 26 and 30); Environment (Items 11,22,24 and 29); Professional training (Items 1,15,17,19,25 and 27) and Theoretical activities (Items 2,10,13,14 and 28). The items are presented in a Likert-type scale with four points, where: zero - “I do not experience this situation”; one - “I do not feel stressed about this situation”; two - “I feel a little stressed about this situation”; and three- “I feel very stressed about this situation”. 

After data collection, the data were inserted in an Excel spreadsheet (Office 2010) and analyzed in the R Statistical Package (Version 3.3.0) and its complement *Lavaan* (*latent variable analysis*), version 0.5-20. Among the selected students, 524 were used in the exploratory factor analysis (EFA) and 523 in the confirmatory factor analysis (CFA). The Kaiser-Meier-Oklin (KMO) and the Bartlett’s test of sphericity were used as measures of adequacy of *the* sample in the EFA; KMO values > 0.50 and p-values < 0.05 in the test of Bartllet’s test were considered adequate ​​for the factorial *analysis*. The extraction of the factors was obtained through parallel analysis, where factors with eigenvalues ​​greater than the eigenvalues ​​obtained with random data are maintained^12^. To explore data, we used the non-weighted least squares method with oblique rotation, of the oblimin type. For investigation of the internal structure adjacent to the group of items, the following methods were used: polychoric correlation (0.5 ≤ r ≤0.7); Comunality (0.4 ≤ r ≤ 0.6); Factorial load (0.4 ≤ r ≤ 0.7), *Cronbach’s* Alfa (0.70 ≤ r ≤ 0.90) and Corrected item-total correlation (0.3≤ r ≤ 0.8)^12^. Items with a factor load of less than 0.4 were initially excluded, and a new EFA was performed with the remaining items. This process was repeated until the smallest possible number of items with satisfactory results were obtained in the aforementioned parameters.

CFA was applied to confirm the internal structure underlying the group of variables found in the EFA. The robust weighted least squares technique was used to explore data, with the following indicators of absolute fit: X^2^ (Fit = > 0.05), standardized X^2^ (Fit = < 3.0); Goodness of Fit Index (GFI) (Fit = > 0.95); and the following indicators of incremental fit: Comparative Fit Index (CFI) (Fit => 0.92) and *Tucker Lewis* Index (TLI) (Fit = > 0.92)^12^. As measure of poor quality of fit, we used: root of the mean square error of approximation (RMSEA) (Fit = r < 0.08 considering CFI > 0.92) and weighted residual mean square root (WRMR) (Fit = < 1.00)^12^ ^-^ ^13^. The Factorial Load (0.4 ≤ r ≤ 0.6) and the Polychoric Correlation (0.5 ≤ r ≤ 0.7) allowed us to evaluate the contribution of each observable variable to the latent variables^12^.

This work is a subproject of the project Stress, *Coping*, Burnout, Depressive Symptoms *and* Hardiness in Nursing Students and Teachers, approved by the Research Ethics Committee (REC) under nº. 0380.0.243.000-10. In compliance with Resolution 466/12 of the National Health Council, a Consent Form was given to the study participants, through which they expressed their authorization of voluntary participation in the study. 

## Results

Initially, there were 1179 nursing students enrolled in the nursing schools. However, four students were not enrolled in professional training subjects, 91 were not present on the day of collection, 3 were in exchange, 27 did not return the instruments in the expected period; three participated in the project as researchers; and four students did not agree to participate in the study. Thus, a population of 1,047 students was obtained, being: 316 of the School of Nursing of the University of São Paulo (EEUSP); 77 of the Federal University of São Carlos (UFSCAR); 136 of the Federal University of Espírito Santo (UFES); 154 of the Federal University of Bahia (UFBA) and 364 of the Paulista University (UNIP - SP).

In the initial exploratory factorial analysis, KMO was found to be 0.87, with significance in the Bartllet’s Sphericity Test (p < 0.001), indicating the possibility of factorization of the instrument. The parallel analysis showed the existence of five factors (explained variance of 43.2%), with items distributed as follows: Factor1 (Items 4, 6, 7, 8 and 12); Factor 2 (Items 2, 3, 10, 13, 14, 21, 23, 26, 28 and 30); Factor 3 (Items 11, 22, 24 and 29); Factor 4 (Items 17 and 18); and Factor 5 (Items 5, 9, 12, 15, 16, 19, 20, 21, 25, 27 and 28). It should be noted that item 1 did not saturate in any factor. Factorial loads ranged from 0.312 to 0.911 and commonalities from 0.114 to 0.778. The Cronbach’s alpha coefficient ranged from 0.743 (Factor 3) to 0.854 (Factor 5) between domains and the corrected item-total correlation ranged from 0.255 (Factor 2) to 0.610 (Factor 4). 

**Figure 1:**
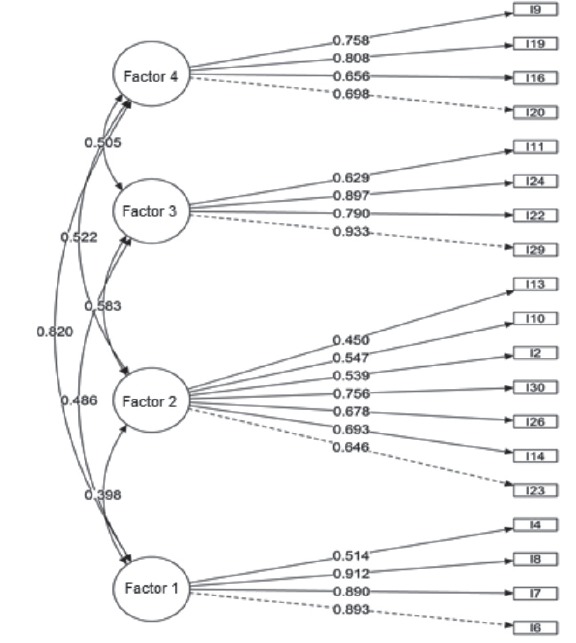
Model of Measurement of the Instrument for Assessment of Stress in Nursing Students (ASNS) - short version. São Paulo, SP, Brazil, 2016

Since items 5 and 21 have factorial loads (0.312 and 0.332 respectively) and commonalities of less than 0.4 (0.316 and 0.259 respectively), both were excluded at the first moment. The items in the same condition were subsequently deleted and the parameters evaluated (KMO, Bartlett’s test, correlations, commonalities and factorials loads etc). In this process, items 1, 3, 12, 15, 17, 18, 25, 27 and 28 were also eliminated, leading to the short version of the instrument (KMO = 0.84, Bartlett’s test < 0.001). This version was composed of 19 items distributed in four factors that explained 53.9% of the total variance. Commonalities ranged from 0.270 to 0.942 and factorial loads from 0.455 to 0.918. Only in the items 2 (H^2^ = 0.285), 10 (H^2^ = 0.291) and 13 (H^2^ = 0.270), the commonalities were below and, in the item 29, above the established limit (H^2^ = 0.942). Correlated item-total correlation values ​​were: 0.572 (Factor 1), 0.419 (Factor 2), 0.285 (Factor 3) and 0.492 (Factor 4). The findings of the confirmatory factor analysis are presented in Figure 1.

In the CFA, it was observed that all the items presented satisfactory factorial loads, indicating that they contribute to explain the latent variables (factors) in question. Correlations among the domains presented satisfactory values and confirmed their interdependent behavior in explaining stress in nursing students.

Factor 1 (Performance of Practical Activities) was composed of four items of the original instrument, namely: 4 - Performance of assistance procedures in general; 6 - Communication with other professionals of the internship unit; 7 - Environment of the clinical internship unit; and 8 - Communication with professionals from other sectors in the internship place. Factor 2 (Theoretical Activities) was composed by seven items, as follows: 2 - Mandatory character of extra-class activities; 10 - Method adopted to evaluate theoretical contents; 13 - Feeling insecurity or fear at theoretical tests; 14 - Degree of difficulty to perform extra-class activities; 23- Deadlines established by teachers for delivery of extra-class activities; 26 - Lack of time for leisure; and 30 - Lack of time for moments of rest. Factor 3 (Environment) was composed by the same four items of the original version: 11 - Distance between college and residence; 22 - Public transportation used to reach college; 24 - Distance between most of internship sites and the residence; and 29 - Public transportation used to reach the internship site. Factor 4 (Professional Training) was composed by four items of the original instrument: 9 - Fear of making mistakes during provision of patient care; 16 - Perception of difficulties in the relationship with other professionals in the area; 19 - Perception of professional accountability when working in the internship; and 20 - Observation of conflicting attitudes in other professionals. 

The values ​​obtained for the absolute measures were: X^2^759.46; standardized X^2^= 5.20; p < 0.001; GFI = 0.98. In parsimony measures, CFI and TLI values were both 0.97. These results were satisfactory except for the standardized X^2^, which confirmed the absolute and parsimonious fit of the proposed model. RMSEA and WRMR values were respectively 0.09 and 1.43, indicating the presence of residues within an acceptable limit. In addition, Cronbach’s alpha coefficient was 0.842 for the Factor Performance of Practical Activities; 0.743 for Theoretical Activities; 0.736 for Environment and 0.795 for Professional Training. These values ​​demonstrate satisfactory reliability of the instrument.

## Discussion

One of the purposes of the factor analysis is to evaluate the dimensionality of a set of indicators in order to identify the least number of factors necessary to explain the phenomenon in question^13^. In this context, the factorial analyses led to a short version of the ASNS with 19 items organized in 4 factors, whose construct validity and reliability were satisfactory for measuring stress in university nursing students.

During the analysis of the internal structure of the instrument, it was observed that the commonalities of items 2, 10 and 13 were below the established limit, and of item 29 was above this limit. The values ​​found for items 2, 10 and 13 indicated that they have low power in the explanation of stress when in conjunction with the other items (common variance)^12^. On the other hand, item 29 presented a possible collinearity; this means that there was a linear relationship between two explanatory variables^12^. However, it is necessary to evaluate the other parameters to confirm these aspects^12^ ^-^ ^13^. As the results obtained on factorial load, item-total correlation and polychoric correlation were satisfactory, the items in question were maintained. 

Satisfactory factorial loads and inter-factor correlations were observed in the investigation of validity of the construct through CFA. All parsimony fit indices and most of the absolute measures presented acceptable values. However, the standardizedX^2^, the WRMR and the RMSEA presented values ​​slightly above expectations. Although above the ideal, similar values ​​of residues were verified in other researches with stress instruments, being considered acceptable by different researchers^2^ ^,^ ^15^. The Chi-square test requires large sample values ​​for confirmatory factor analysis, which explains the sensitivity of this indicator to the sample size. Therefore, evaluating the other indicators concomitantly before changing the model is advisable^13^. The observation of the other parameters altogether confirmed the construct validity of the instrument. This type of validity refers to the ability of an operational definition (construct) to truly reflect the theoretical meaning of a given concept^16^. Therefore, after obtaining the final structure, each factor of the instrument was redefined based on the items that composed it.

 Thus, the factor Performance of Practical Activities evaluates the difficulties related to the clinical environment, including performing procedures and communicating with health professionals^2^. The presence of items related to professional communication in this factor is justified by the fact that, during practical activities, students are exposed to the need to communicate with staff professionals and patients^2^. This, in turn, involves the application of technical terms, technical-scientific knowledge and interpersonal relationship skills^11^ ^,^ ^17^, elements that can be perceived as stressors by students in view of their inexperience in the care field^18^. In this sense, communication is an element that helps to explain the stress experienced by the student during the performance of Practical Activities. The factor Theoretical Activities encompasses items that measure students’ stress caused by theoretical tests; the evaluation method of programmatic content; deadlines for delivery of extra-class activities; and the conciliation of these aspects with other personal, social and emotional responsibilities and demands. The three items related to time management were considered to fit in this factor because, faced with the difficulty of reconciling academic and personal activities, students put more effort on the former, exceeding their cognitive resources and this contributes to the stress in dealing with theoretical activities^2^ ^,^ ^11^. 

The Factor Environment contains the same four items of the original version (11, 22, 24 and 29), with no structural changes in relation to the original instrument. This factor measures the stress related to the difficulty to access the internship and/or university fields; and those related to the use of means of public transportation^2^, frequent problems in urban centers and that interfere in the daily life of students, causing stress. The Factor Professional Training involves the perception of risks involved in providing patient care; the professional accountability in the field of internship; difficulties in the interactions with the team; and conflicting attitudes towards other professionals. Authors have confirmed that relationships among nursing professionals are influenced by the daily routine of care and the work environment, contributing to stress^17^. The contact with the assistance allows the student to experience challenges inherent in the nurses’ performance, leading to feelings of insecurity regarding their professional training.

In the short version , Cronbach’s Alpha values ranged from 0.736 (Factor 3) to 0.842 (Factor 1). The corresponding factors presented similar values ​​in the original version: 0.806 (Performance of Practical Activities), 0.866 (Environment), 0.772 (Professional Training), 0.720 (Theoretical Activities)^2^. These values ​​attest a satisfactory reliability of the short version of the ASNS, evidencing its ability to produce equivalent results after different applications^12^ ^,^ ^16^. 

**Figure 2:**
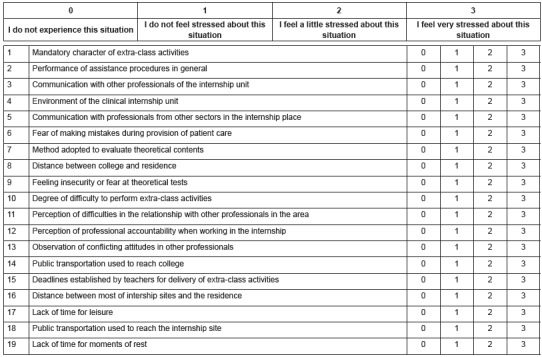
Final Version of the Instrument for Assessment of Stress in Nursing Students (ASNS) - SHORT VERSION

The ASNS - Short version consists of 19 items with a likert-type scale of four points distributed into four domains: Performance of Practical Activities (Items 2, 3,4 and 5); Theoretical Activities (Items 1,7,9,10,15,17 and 19); Environment (Items 8, 14, 16 and 18); and Professional Training (Items 6, 11, 12 and 13) (Figure 2).

## Conclusion

The short version of the instrument has construct validity and reliability to be applied to Brazilian nursing students at any stage of the course. This instrument has a simpler structure, which favors the adherence of participants and its use by researchers. Considering that the construct validity is strengthened as the instrument is used by researchers, it is suggested that this version be applied in future samples of nursing students so as to evaluate its psychometric properties and make eventual necessary corrections.

The intention of the version proposed here is to evaluate the level of stress of nursing students at any stage of the course. In this sense, the application of the ASNS - short version in students of a specific year of the course may lead to low scores in one or more domains because the curricula of most institutions prioritize theoretical activities at the beginning of the course, and practical activities at the end. For this degree of specificity, it would be important to adapt the instrument to each phase of the course. Furthermore, although the construct validity of the ASNS - reduced version was confirmed, the evaluation of its validity of criteria is still necessary in future studies to attest its full validity.
